# Cardiff Mental Hospital

**Published:** 1920-11-06

**Authors:** 


### Cardiff Mental Hospita .

THE impending retirement of Alderman Morgan Thomas from the chairmanship of the Cardiff Mental Hospital Visiting Committee will close a long record of public work. Mr. Morgan Thomas has been connected with the administration of the hospital for about twenty-five years, and was the first chairman of the Mental Hospital Committee. Under the medical superintendentship of Dr. Edwin Goodall, the institution has become a hospital instead of a place of detention only. To illustrate this, Mr. Morgan Thomas, at the last meeting of the Committee, referred to a patient who was discharged cured after having been bedridden for six years in other institutions. He spoke also of his retirement, and referred to the loyal support which he had always received from his colleagues.

Mr. Morgan Thomas presided at the first two national conferences on the amendment of the Lunacy Acts at the Guildhall in London, and for two years was President of the National Mental Hospital Association. He was Lord Mayor of the city in 1912-13, and during the war became recruiting officer for Tredegar, Merthyr, and Rhondda districts. After three years in the Army, retiring with the honorary rank of captain, he became Assistant Director under the Ministry of Food, a position which he resigned last February. In the political and religious activities of Wales he has also taken an active part.

